# Challenges in molecular diagnosis of multiple endocrine neoplasia

**DOI:** 10.3389/fendo.2024.1445633

**Published:** 2024-09-27

**Authors:** Pauline Romanet, Théo Charnay, Nicolas Sahakian, Thomas Cuny, Frédéric Castinetti, Anne Barlier

**Affiliations:** ^1^ Aix Marseille Univ, APHM, INSERM, MMG, La Timone University Hospital, Laboratory of Molecular Biology GEnOPé, BIOGENOPOLE, Marseille, France; ^2^ Aix Marseille Univ, APHM, INSERM, MMG, La Conception University Hospital, Department of Endocrinology, Marseille, France

**Keywords:** MEN1, MEN2, genetic testing, candidate gene, genome, mosaicism

## Abstract

Multiple endocrine neoplasia (MEN) is a group of rare genetic diseases characterized by the occurrence of multiple tumors of the endocrine system in the same patient. The first MEN described was MEN1, followed by MEN2A, and MEN2B. The identification of the genes responsible for these syndromes led to the introduction of family genetic screening programs. More than twenty years later, not all cases of MENs have been resolved from a genetic point of view, and new clinicogenetic entities have been described. In this review, we will discuss the strategies and difficulties of genetic screening for classic and newly described MENs in a clinical setting, from limitations in sequencing, to problems in classifying variants, to the identification of new candidate genes. In the era of genomic medicine, characterization of new candidate genes and their specific tumor risk is essential for inclusion of patients in personalized medicine programs as well as to permit accurate genetic counseling to be proposed for families.

## 1 Introduction

Multiple endocrine neoplasia (MEN) is a group of diseases characterized by the occurrence of multiple tumors of the endocrine system in the same patient. MENs are rare genetic diseases resulting from germline genetic defects in a variety of tumor suppressor genes or oncogenes. MENs are hereditary diseases, transmitted in an autosomal dominant manner. The first MEN described was MEN1 (OMIM 131100) (1), followed by MEN2A (OMIM 171400) and MEN2B (OMIM 162300). Initially these syndromes were described clinically, followed by the discovery of underlying genetic causes. Identification of these disease-specific genes has enabled targeted genetic testing of index cases, and in the event of positive results, presymptomatic screening of their relatives. Presymptomatic genetic testing is a crucial aspect of personalized medicine in tumor predisposition syndromes as it enables the identification of carriers and non-carriers of pathogenic or likely pathogenic variants in a family before they show any clinical signs or symptoms of the condition (2). Family members who carry such variants can then benefit from a disease-specific monitoring program, while non-carriers can be reassured and excluded from monitoring. However, a genetic basis is not identified in all MEN patients. More recently, advances in medicine, scientific knowledge and sequencing technologies have revolutionized our conception of MENs, enabling the discovery of new entities such as MEN4 (OMIM 610755) (3) and the resolution of failures in molecular diagnosis. With advances in genomic sequencing technology, the number of gene-disease relationships that have been described has rapidly expanded, though their application in clinical settings is sometimes uncertain.

## 2 MEN syndromes

### 2.1 MEN1

Multiple endocrine neoplasia type 1 results from an inactivating heterozygous mutation in the *MEN1* tumor suppressor gene (**Table 1**) (1). *MEN1* (location 11q13.1) encodes MENIN, a nuclear scaffold protein that is involved in histone modification and epigenetic gene regulation (4). Indeed, the MENIN is an essential component of the MLL/SET1 histone methyltransferase (HMT) complex, a complex that specifically methylates ‘Lys-4’ of histone H3 (H3K4) (5, 6). H3K4 trimethylation is associated with activated gene transcription. A loss of MENIN results in transcriptional repression of specific genes due to loss of H3K4me3 in the promoter. MENIN also functions as a transcriptional regulator, binding to the *TERT* promoter and repressing telomerase expression. It interacts with SMAD3 or SMAD1/SMAD5 to promote their transcriptional activity, and the loss of MENIN in these interactions inhibits the TGF-β and BMP signaling pathways, respectively, thus antagonizing their proliferation-inhibitory action (7). MENIN also represses JUND-mediated transcriptional activation (8). JUND is a member of the JUN family, and a functional component of the AP1 transcription factor complex. It has been proposed that JUND protects cells from p53-dependent senescence and apoptosis. MENIN positively regulates *HOXC8* and *HOXC6* expression and may be involved in normal hematopoiesis through the activation of *HOXA9* expression. In MEN1, patients are predisposed to develop tumors by inheritance of a heterozygous inactivating mutation, while it is complete loss of MENIN or inactivation by a somatic second hit on the other allele that promotes tumorigenesis, according to the two hit Knudson hypothesis (9). MEN1 prevalence is estimated at between 1/10,000 and 1/30,000. The classic clinical triad observed in MEN1 includes primary hyperparathyroidism (PHPT), pituitary neuroendocrine tumors (PitNETs), and duodeno-pancreatic neuroendocrine tumors (DPNETs). Other endocrine tumors, including adrenal cortical tumors, and neuroendocrine thymic or bronchopulmonary tumors, may also be present. Several non-endocrine manifestations have also been reported to be associated with MEN1: angiofibromas, collagenomas, lipomas, and meningiomas. Twenty-eight to 70% of patients with MEN1 die as a consequence of the disease, particularly due to the grade neuroendocrine tumor (NET) lesions (10, 11). MEN1 diagnosis is classically made where any of three different criteria are met: i) the presence of 2 MEN1-related major lesion in one patient, ii) the presence of MEN1-related lesion in a patient with a first degree relative with MEN1, iii) the presence of a MEN1 pathogenic or likely pathogenic variant in a patient, which may or may not be symptomatic (12). An important effort has been made by the international research community to provide clinical practice guidelines for monitoring and genetic testing, and these are currently under revision (for most recent version see (12)).

**Table 1:** Multiple endocrine neoplasia syndromes.

Syndrome	Gene	Gene type	Transmission	Phenotype
MEN1	*MEN1*	tumor suppressor	autosomal dominant	Primary hyperparathyroidism, pituitary NET, duodeno-pancreatic NET, lung NET, thymic NET, adrenal tumor, lipomas, angiofibromas, collagenomas, hibernomas, leiomyomas, central nervous system tumors, breast cancer
MEN2A	*RET*	oncogene	autosomal dominant	Medullary thyroid carcinoma, pheochromocytoma, primary hyperparathyroidism
MEN2B	*RET*	oncogene	autosomal dominant	Early onset medullary thyroid carcinoma, pheochromocytoma, intestinal ganglioneuromatosis, Marfanoid habitus, alacryma, mucosal neuromas
MEN4	*CDKN1B*	tumor suppressor gene	autosomal dominant	Primary hyperparathyroidism, pituitary NET, duodeno-pancreatic NET, adrenal tumor, thymus tumor, papillary thyroid cancer

NET, neuroendocrine tumor.

### 2.2 MEN2

Multiple endocrine neoplasia type 2 occurs due to recurrent heterozygous activating mutations in the proto-oncogene *RET* (**Table 1**). *RET* (location 10q11.21) encodes RET, a transmembrane receptor and member of the tyrosine protein kinase family of proteins. Binding of ligands such as GDNF (glial cell-line derived neurotrophic factor) and other related proteins to the encoded receptor stimulates receptor dimerization, RET intracellular transphosphorylation, and activation of downstream signaling pathways that play a role in cell differentiation, growth, migration and survival. MEN2 prevalence is estimated at approximately 1/35,000. MEN2 was initially separated into three syndromes: MEN2A (95% of MEN2), MEN2B (also called MEN3), and familial medullary thyroid carcinoma (FMTC), due to very specific clinical presentations that depend on the mutations present. Indeed, MEN2 is characterized by a strong genotype-phenotype correlation (13). Major clinical manifestations of MEN2 include medullary thyroid carcinoma (MTC), pheochromocytoma (PHEO) and, in the case of MEN2A, primary hyperparathyroidism (pHPT). The term FMTC has been gradually abandoned in favor of MEN2A, with the identification of low-frequency lesions associated with MTC. Here too, an important effort has been made to provide clinical practice guidelines for monitoring and genetic counseling (13, 14).

MEN2A is mainly caused by activating mutations in exons 10 and 11, and is characterized by variable risks of aggressive MTC, pheochromocytoma, PHPT or cutaneous lichen amyloid depending on the mutation (14). Mutations in exons 10 and 11 affect cysteines in the extracellular domain of the RET receptor. They cause dimerization of receptor molecules, enhanced phosphorylation and thus ligand-independent activation of intra-cellular pathways. Mutations in codon Cys634 (exon 11) are associated with a high risk of aggressive MTC as well as a very high risk of pheochromocytoma (about 50%) and, to a lesser extent, of PHPT. MEN2A *RET* mutations in exon 10 can be associated with Hirschsprung’s disease, a rare congenital intestinal motility disorder, due to the presence of an aganglionic segment in the terminal part of the colon. MEN2A variants in the intracellular tyrosine kinase domain are less frequent (exons 13 to 16). These are associated with a milder risk of PHTP and pheochromocytoma, except for the M918T variant in exon 19 and the A883F variant in exon 15 that cause MEN2B.

MEN2B is mainly due to a specific activating mutation c.2753T>C, p.Met918Thr (M918T). This change from a methionine to threonine within the activation segment of RET kinase, increases ATP-binding and auto-phosphorylation activity, thereby mediating a dimerization-independent activation of RET kinase. MEN2B is characterized by early and aggressive medullary thyroid carcinoma (MTC), pheochromocytoma, mucosal neuromas, and thickened corneal nerves. Most affected individuals have characteristic physical features, including full lips, thickened eyelids, high-arched palate, and marfanoid habitus (long arms, long legs, arachnodactyly). Less frequently affected individuals present skeletal anomalies and gastrointestinal problems (15).

### 2.3 MEN4

Multiple endocrine neoplasia type 4 (MEN4) results from heterozygous inactivating mutations in the *CDKN1B* tumor suppressor gene (**Table 1**) (3). *CDKN1B* (location 12p13) encodes P27KIP1 which is a cyclin-dependent kinase inhibitor that is normally activated by MENIN (encoded by *MEN1*) to negatively regulate the cell cycle and limit G1-S phase transition. MEN4 was identified in human by its homology with a mouse model that develops MEN1-related lesions. The prevalence of MEN4 is unknown, but is lower than MEN1, with MEN4 patients developing a MEN1-like phenotype with a later onset and with incomplete penetrance (3, 16). To date, there is no consensus on genetic testing, monitoring, and genetic counseling for MEN4.

## 3 MENs -diagnostic challenges and pitfalls

### 3.1 General strategy for molecular diagnosis of MENs:

In practice, patients with clinical or suspected MEN may benefit from germline genetic testing targeting the disease-specific gene. Traditionally, genetic tests in index cases targeted a single gene and were performed using the Sanger method. The Sanger method is a DNA sequencing method based on the random incorporation of chain-terminating fluorescent dideoxynucleotides by DNA polymerase during *in vitro* DNA replication, that are detected by electrophoresis (17). More recently, the Sanger method was abandoned in favor of new methods that enable several genes to be examined at the same time, known as next generation sequencing (NGS) procedures (18). NGS platforms perform sequencing of millions of small fragments of DNA, called reads, in parallel. NGS rapidly became preferred over the Sanger method for genetic testing of index cases because i) NGS captures a broader spectrum of mutations than Sanger sequencing, from small base changes (substitutions) to large genomic deletions of exons or whole genes (which Sanger sequencing cannot do); ii) NGS is cost effective, allowing the sequencing of several genes, up to the whole genome, in several samples, simultaneously.

**Figure 1:**
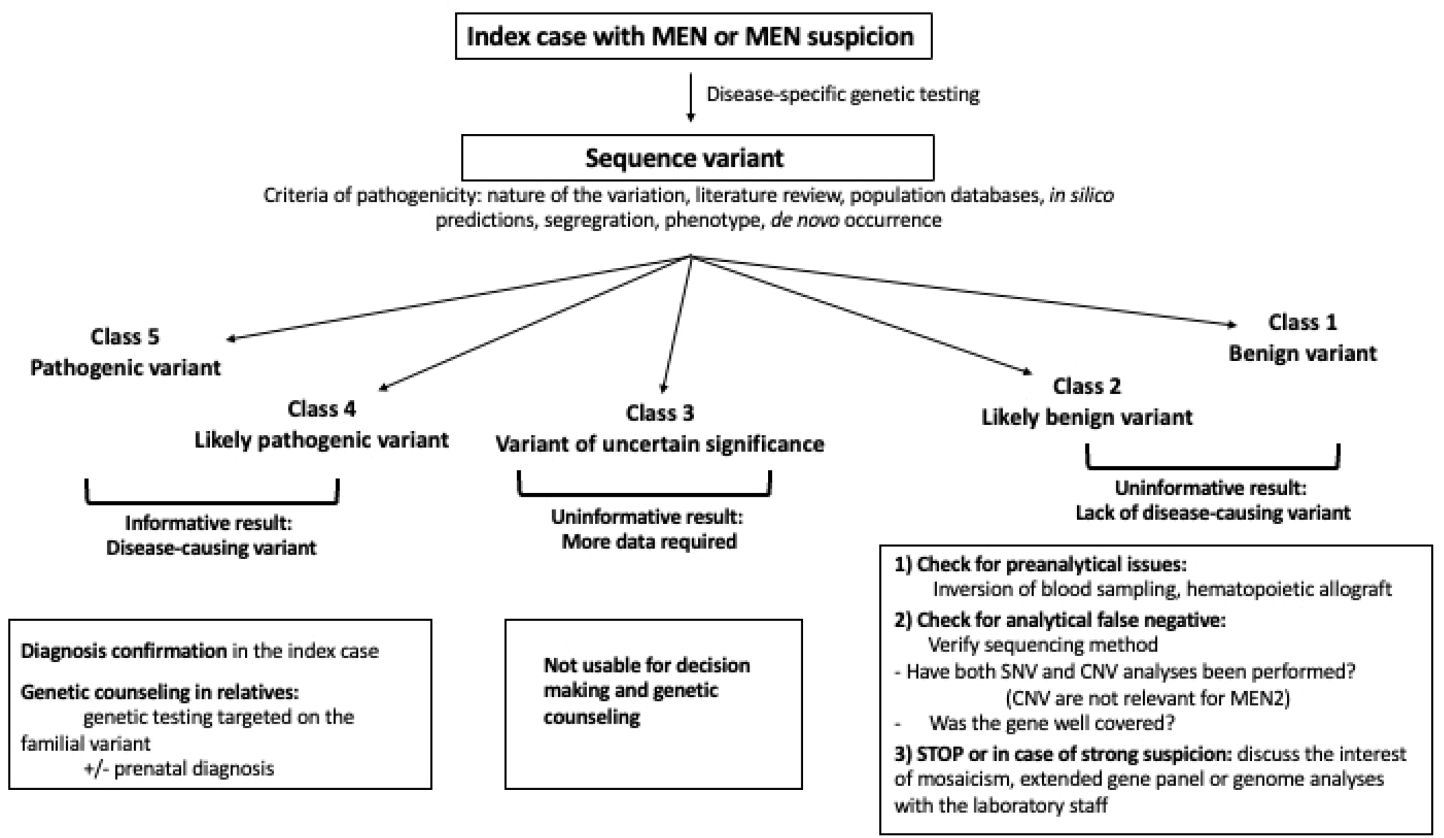
Flow chart of genetic testing, interpretation and use of sequence variants in a clinical context. CNV, Copy number variation; SNV, single nucleotide variation.

After sequencing, variants are classified into 5 classes of pathogenicity: class 1: benign variant (BV), class 2: likely benign variant (LBV), class 3: variant of uncertain significance (VUS), class 4: likely pathogenic variant (LPV), class 5: pathogenic variant (PV, see **Figure 1** (19). The use of this classification system allows medical care to be adapted according to the likely effect of the variation. For a patient with a disease, a BV or a LBV in a gene is considered as not causing or likely to not cause the disease, while a PV is considered to be responsible for the disease. In the case of a LPV, this means that there is enough evidence to consider that the variant causes the disease, but there is still a certain degree of uncertainty. Consequently, this information should be used with caution for clinical decision-making. In the case of PV or LPV, the test is considered as positive and the diagnosis is confirmed, even in patients with an incomplete phenotype. Moreover, their relatives can benefit from genetic counseling, including presymptomatic screening. Pre-symptomatic screening is based on a targeted search of the familial anomaly, generally by Sanger sequencing looking for single nucleotide variations (SNV) and MLPA for copy number variations (CNV).

The next category, VUS, consists of variants suspected of causing the disease, but with insufficient or conflicting evidence. The distinction between LPV and VUS is very important because the presence of a LPV can be used not only for decision making regarding patient care, but also for genetic counseling, including the testing of family members, prenatal diagnosis etc (20). On the other hand, a VUS should never be used for decision making and genetic counseling. Nevertheless, appropriate follow-up of patients with variants classed as VUS is necessary to check for potential re-classification, which may clearly impact on clinical decision making and subsequent familial genetic counseling.

### 3.2 MEN1

In MEN1, classical genetic testing on blood DNA is positive in 90% to 95% of familial cases, and in 30-45% of sporadic cases who present with the classical triad (21–23). In patients with 2 MEN1 major lesions, the mutation detection rate is less than 20%, depending on the associated lesions. Nevertheless, the identification of a *MEN1* pathogenic variant is crucial since even though the MEN1 diagnosis can be done on clinical basis, identification of the pathogenic variant is required to inform the genetic counseling, meaning familial presymptomatic genetic testing and in some cases, allowing prenatal diagnosis to be proposed. In MEN1, presymptomatic screening allows a genetic diagnosis to be made, and to then include the MEN1-genetically positive member of the family in a specific follow-up program. For MEN1-genetically negative members, the risk of having MEN1 is then considered equal to the risk in the general population, i.e. a low risk according to the prevalence of the disease, hence they are excluded from specific management. Conceptually, in the absence of identification of *MEN1* pathogenic or likely pathogenic variants in a patient with a clinical or familial diagnosis of MEN1, all of the family members must be considered at risk and be included in the MEN1-specific follow-up program.

*MEN1* inactivating mutations can occur throughout the gene sequence. In the UMD-MEN1 database of French patients with *MEN1* variants, microinsertions or deletions have been identified in half of the index cases and missense substitutions in one quarter of cases (24). Splice junction, mid-intronic, and synonymous substitutions are less frequent. Copy number variations (CNV), i.e. large deletions, affecting one or several exons were found in 2.2% of index cases with a *MEN1* variant. The genotype-phenotype relationship remains a matter of debate in MEN1, except for variants affecting the JUND interacting domain (25).

An important question that arises is, since the mutation detection rate is inferior to 20% in index cases with two major lesions of MEN1-2major lesions, do all of the family members need to be included in the MEN1-specific follow-up program if no pathogenic or likely pathogenic variant is identified by genetic testing.

Faced with a genetically-negative MEN1 patient, the first step is to verify that both single nucleotide variations (SNV) and copy number variations (CNV) were searched for during genetic testing (26). CNVs most frequently affect one or more *MEN1* coding exons, however a heterozygous 596bp deletion between nucleotides -1087 and -492 upstream of the translation start site has recently been described, located within the 5’ untranslated region (UTR) of *MEN1*, and including the core promoter and multiple cis-regulatory regions (27).

Variations in the deep intronic or promotor regions may also cause MEN1 by altering *MEN1* splicing or expression level. Nevertheless, genome wide analysis can be used to rectify false negative MEN1 genetic testing. For example, Backman et al. sequenced the constitutional genome of fourteen patients with a clinical diagnosis of MEN1 (n = 13) or suspected MEN1 (n = 1) who had negative first-line MEN1 genetic screening (28). They found that three patients carried *MEN1* pathogenic variants (two splice-site variants, one missense variant) that had not been detected during routine clinical sequencing. Analytical false negatives are a side effect of the paradigm change between Sanger sequencing and next generation sequencing (NGS).

Clinical Sanger sequencing requires a manual review of the sequences, which is time-consuming but relatively simple for heterozygous or homozygous variant identification, but requires specific expertise for the detection of unconventional abnormalities such as mosaicism. On the other hand, NGS generates a large amount of data that are mainly treated in an automated manner. Consequently, numerous precautions must be taken using bioinformatics tools to ensure the quality and accuracy of the sequencing. Among these, sequencing depth and coverage are two crucial concepts. Sequencing depth refers to the number of times a specific base in the DNA sequence is read during the sequencing process. For germline analysis, it is generally recommended that a given position is sequenced a minimum of 20 or 30 times to accept that the targeted sequence has been effectively sequenced. This data is assessed by the coverage, the proportion of the target sequence that was sequenced at a certain depth. Less than 100% coverage for a gene of interest may mean that a variant was missed because it was not effectively sequenced. Custom targeted gene panels are most often designed to precisely target the coding sequences and exon-intron junctions of the genes of interest, and thus avoid coverage defects. This is particularly well-suited to the study of specific diseases such as the predisposition to endocrine tumors. It is not always the case with comprehensive hereditary cancer gene panels or whole exome sequencing approaches, which may be chosen by general hospitals to meet the needs of different specialists in a variety of diseases (29).

**Figure 2:**
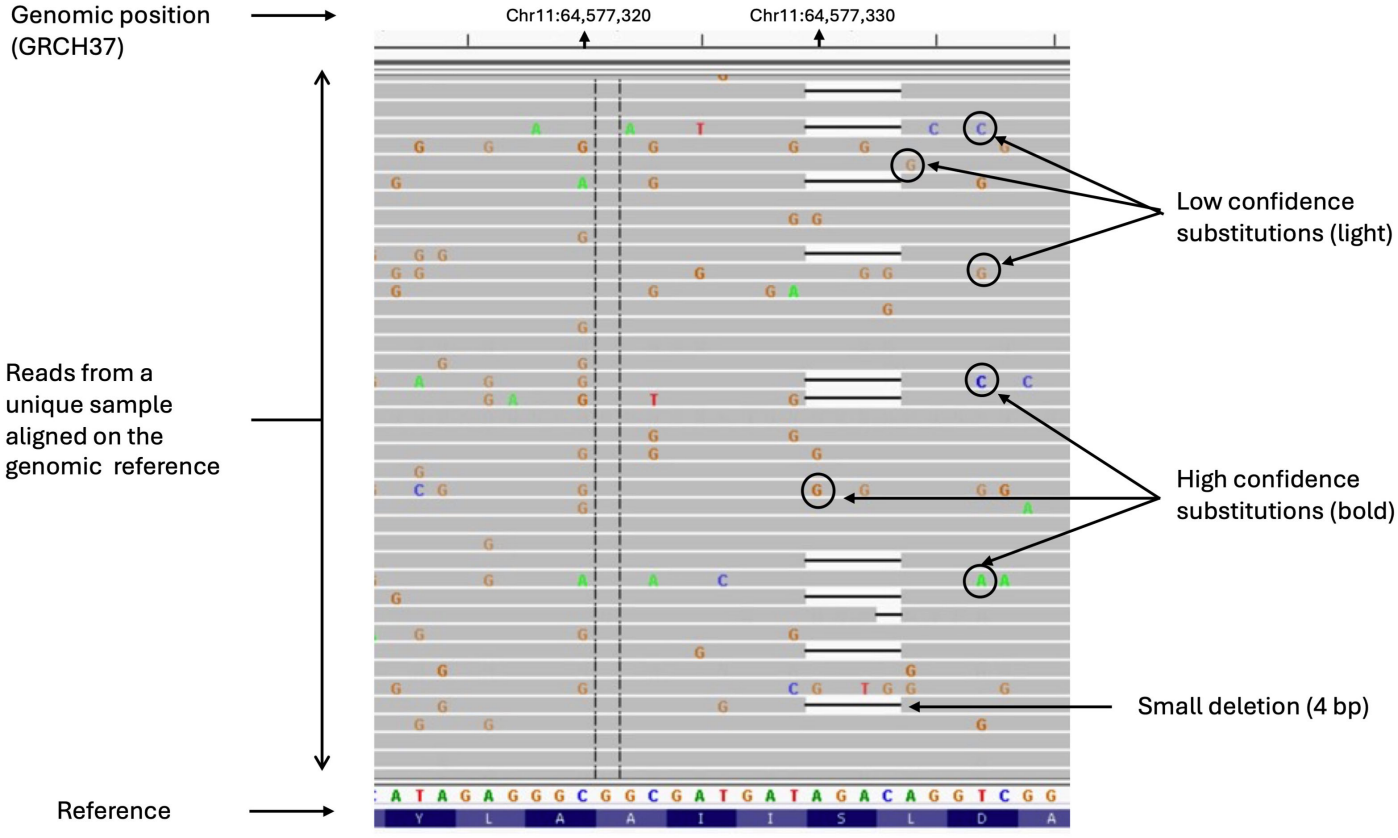
Read alignments produced on an Illumina MiSeq sequencer and visualized using IGV 2.11.3. The reads show a GC-rich region in the second exon of the *MEN1* gene. The top bar indicates chromosomal coordinates. Bold letters indicate mismatch bases corresponding to putative variants. Color intensity indicates the sequencing quality values. Clear letters denote poor sequencing quality scores. In these regions, the quality of sequencing is suboptimal leading to potential false-positive results. To avoid this, variants are filtered out based on the quality value, which can also lead to rejection of a true variant if its quality is insufficient. Here the deletion c.249_252del heterozygous of four bases (indicated by the horizontal black line) was filtered out in first-line analysis because the quality value attributed to an absence of signal (deletion) is the quality value of the surrounding region.

Variants are extracted by matching the reads, i.e. sequences, against the reference genome, and filtered to avoid background noise and recurrent artifacts. This filtering is based on the mutant allele frequency of the variants, on the coverage, but also on data quality, such as quality value (QV). Indeed, higher sequencing depth provides more confidence in the accuracy of the base calls at that position and helps to reduce sequencing errors and noise. Moreover, during the NGS process, a QV is assigned to each nucleotide in a read. These QVs express the confidence that the corresponding nucleotide call is correct (30). When sequencing quality reaches Q30, virtually all the reads will be perfect, with no errors or ambiguities. This Q30 score is considered a benchmark for quality in NGS, and is frequently used for filtering out potential false positive variants. Unfortunately, some regions of the genome may still be difficult to sequence, such as GC-rich regions that are frequently found at the beginning of genes, including *MEN1* (**Figure 2**). In these regions, the QV of a true pathogenic variant may be inferior to the threshold and may not pass the filters, leading to a false negative result which can be rectified either by using more permissive bioinformatics analysis or by changing the sequencing technology. Nevertheless, in their genome study, other than the three patients with a pathogenic variant in the coding sequence of *MEN1*, Backman et al. also identified one patient carrying a pathogenic variant in *CASR* and one patient carrying a gross deletion on chromosome 1q which included the *CDC73* gene. Finally, in six patients without mutations, analysis of matched tumor DNA did not detect any recurrent genes fulfilling Knudson’s two-hit model. These data showed that deep intronic or promoter pathogenic variants are probably not frequently found in genetically-negative MEN1 patients.

In parallel with the evolution of knowledge about MEN1, other genetic diseases that can give confounding phenotypes, such as MEN4, familial PHPT due to *CDC73* inactivating mutations (31), familial hypocalciuric hypercalcemia type 1, 2, and 3, respectively due to *CASR*, *GNA11*, or *AP2S1* inactivating mutations (32–34), and familial PitNET due to *AIP* (35, 36) inactivating mutations, have been discovered. When faced with patients presenting with isolated MEN1-related lesions at a young age or with atypical clinical presentations, it is necessary to screen not only for variants in *MEN1* but also in other genes that are known to be involved in confounding phenotypes. This has now been made possible thanks to the availability of high-throughput sequencing methods in diagnostic laboratories, even for targeted analysis.

**Figure 3:**
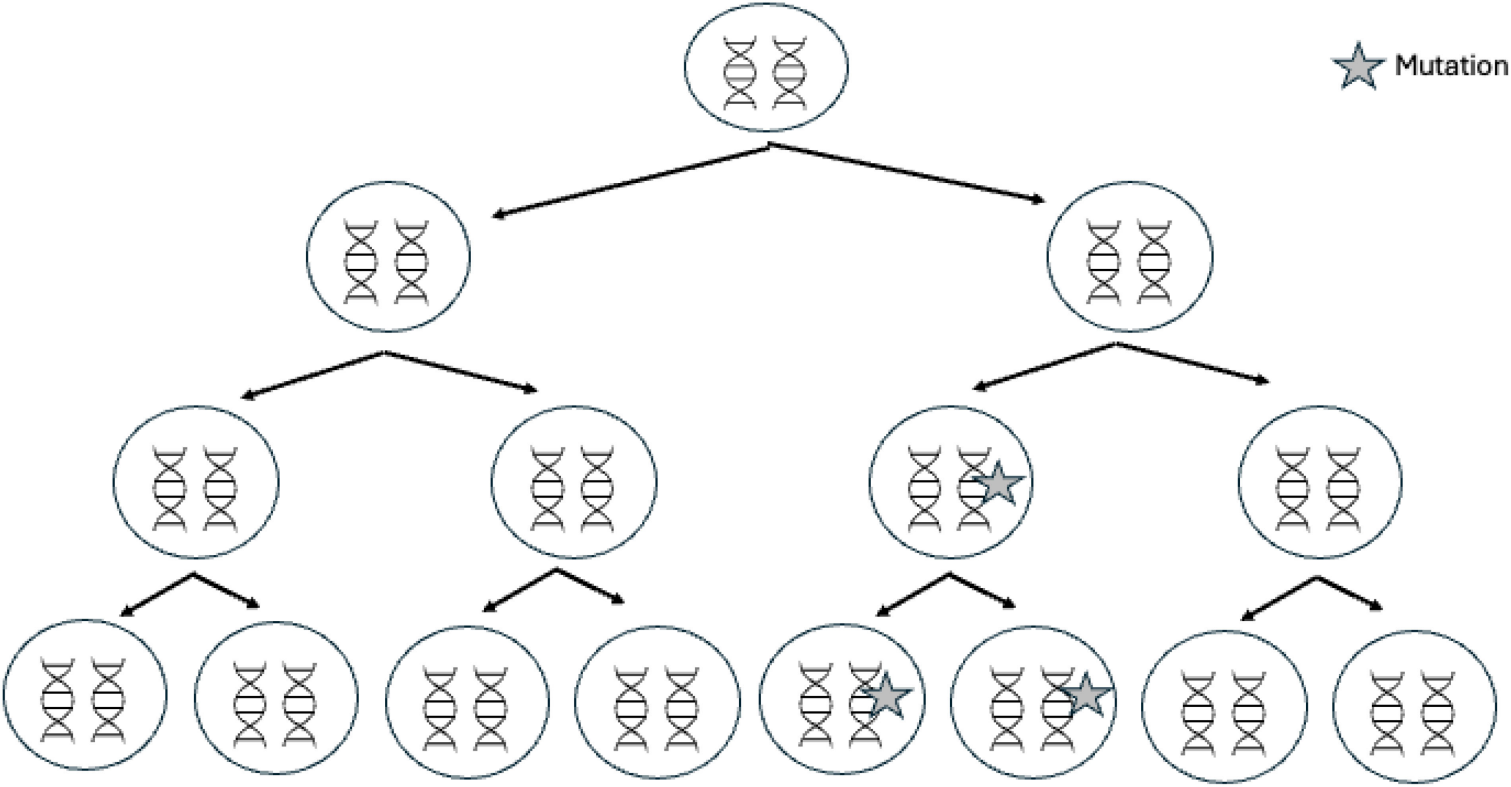
Genetic mosaicism. Mosaicism is due to the occurrence of a *de novo* mutation during postzygotic development, after fertilization. In these cases, only a proportion of the cells will harbor the variation; this feature is termed mosaicism or somatic mosaicism.

Mosaicism can explain some of the unresolved MEN1 cases (37–39). Mosaicism corresponds to the spontaneous acquisition of a genetic variant during cell division occurring in post-zygotic embryonic development (**Figure 3**). Mosaicism thus results in a fetus, and then an individual, composed of a variable proportion of cells carrying the mutation, depending on how early and in which cell lines the variant occurs. Mosaic variants may be undetectable in blood samples using classical sequencing methods because the mutated allelic frequency is too low; indeed levels of mosaicism below ∼10%–20% are difficult to reliably detect using Sanger sequencing (40).

Only a few cases have been reported to date and the frequency of *MEN1* mosaicism is probably underestimated (38). Indeed, identification of MEN1 mosaicism remains challenging in routine practice in diagnostic laboratories and consequently it is not systematically searched for. Moreover, as *MEN1* mutations can occur along the entire length of the gene, it is not possible to use a sensitive targeted method such as digital droplet PCR in first line. NGS offers new possibilities for detecting mosaic variants (41). However, low-frequency variants are often filtered out as they are difficult to distinguish from background noise in bioinformatics pipelines. In a previous study, we evaluated the performance of NGS with unique molecular identifiers (UMI) in the diagnosis of *MEN1* mosaicism in routine practice. UMIs are unique oligonucleotide sequences which are added to DNA prior to any amplification and they differentially label each molecule in the native DNA fragment. UMIs allow for a computational correction of amplification bias and sequencing errors that improves molecular detection of rare events. Among a cohort of 119 patients harboring from 2 to 5 MEN1-related lesions, we identified 3 patients with *MEN1* mosaic pathogenic variants. The mutated allele frequencies ranged from 2.3 to 9.5%. The detection rate of *MEN1* mosaicism in patients bearing at least 3 MEN1 lesions was 17% (3/18). No cases were detected in patients with 2 lesions.

The last aspect affecting accurate MEN1 molecular diagnosis is the assessment of the pathogenicity of *MEN1* variants. In the last ten years, significant efforts have been made to standardize and harmonize the criterion for classification of sequence variants (42). The main benefit is an improvement in the robustness of classification, from expert opinion to evidence-based medicine. For many years, the Human Genome Variation Society and others scientific groups have promoted the use of the 5 pathogenicity classes to describe variants identified in genes that cause Mendelian disorders. This classification reflects the diversity of the human genome and the state of scientific and medical knowledge. In 2015, the ACMG-AMP Variant-Interpretation Guidelines revolutionized the evaluation of sequence variants in human Mendelian diseases (42). These guidelines provide a general procedure for an evidence framework including 28 classification criteria divided into 8 evidence types: “population data”, “computational and predictive data”, “functional data”, “segregation data”, “*de novo* data”, “allelic data”, “other database” or “other data” (including clinical data). Each criterion is weighted for a benign effect (stand-alone evidence of benign impact: BA; strong evidence of benign impact: BS, supporting evidence of benign impact: BP) or a pathogenic effect (very strong evidence of pathogenicity: PVS; strong evidence of pathogenicity: PS; moderate evidence of pathogenicity: PM; supporting evidence of pathogenicity: PP), for examples: criterion PM1 (moderate evidence of pathogenicity): variant located in a mutational hot spot and/or critical and well-established functional domain (e.g., active site of an enzyme) without benign variation, criterion BS3 (strong evidence of benign impact): variant with well-established *in vitro* or *in vivo* functional studies showing no deleterious effect on protein function or splicing. The compilation of evidence scores led to more objective and reproducible classification of variations into one of the five classification classes (examples: 1 PVS criterion + 1 PS criterion or 1 PS criterion + 3 PM criteria lead to a PV classification, 2 PM+ 2 PP: LPV classification, 2BS: LBV). When the criteria for benign or pathogenic variant are insufficient or contradictory, the variant is classified as a variant of uncertain significance.

We have observed that the ACMG-AMP classification leads to an over-classification of variants as VUS compared to the classification by a consortium of experts, especially for *MEN1* missense variants, which is the reason we have proposed adjustment to this classification for *MEN1* missense variants (43).

On the other hand, the ACMG-AMP has also made it possible to reclassify variants, wrongly considered as pathogenic, into benign or likely benign variants. For example, the variant NM_130799.2(*MEN1*):c.1618C>T, p.(Pro540Ser) was subsequently classified as pathogenic, likely pathogenic, and variant of uncertain significance in different studies (44–46). A comprehensive study, including general population data, family co-segregation study, and tumor loss of heterozygosity analysis finally led to this variant being classified as benign (47). For patients, if MEN1 is still suspected based on clinical data, even in the case of a first-round negative genetic result, genetic analysis should be continued, for example by searching for CNV or mosaicism.

### 3.3 MEN2

There are few pitfalls in the diagnosis of MEN2 since MEN2 results from well-known recurrent punctual variations. The identification of a *RET* mutation at the somatic or germline level is essential since RET inhibitors are now used in the management of MTCs. If a MEN2-related pathogenic variant is identified in a MTC at the somatic level, it is important to check at the germline level to rule out MEN2. Indeed, MTC is due to MEN2A in 16% of cases (48).

### 3.4 MEN4

*CDKN1B* variants are less frequently identified in MEN1-suspected patients than *MEN1* variants (16). Nevertheless, the variant interpretation is subject to the same issues, especially the low and late penetrance of the disease making familial segregation studies difficult to interpret. On the other hand, several teams have developed *in vitro* analyses based on the nuclear localization of the P27^KIP1^ protein, encoded by *CDKN1B*, the degradation kinetics of the protein, or based on the molecular function of this protein (49–53). Unconventional variants may also be identified in MEN4 patients. Indeed, in 2013 Occhi and al identified a variant located in the 5’ untranslated region (5’UTR) of the *CDKN1B* mRNA, in a highly conserved regulatory upstream open reading frame (uORF). This variant, creates a frameshift and elongation of this uORF, leading to reduced P27^KIP1^ activity.

## 4 New MENs

The association between PA and PPGL was first described by Iversen in 1952, and can occur in clinical MEN1 or independently (54). The condition, termed the “3PAs” syndrome (for PitNET/pheochromocytoma/paraganglioma association) by Xekouki, can be described as the co-occurrence of PA and PPGL without other features of MEN1 syndrome (55). This association is rare, with fewer than 200 cases published to date. In half of the cases, an association with a genetic variant has been reported, mainly in genes involved in predisposition to pheochromocytoma and paraganglioma such as *SDHA* (OMIM 614165)*, SDHB* (OMIM 115310)*, SDHC* (OMIM 605373) and *SDHD* (OMIM 168000) genes coding for the subunits of succinate dehydrogenase, and *MAX* (OMIM 171300) (46, 55–59). Though the association between *SDHx* variants and PitNETs seems to be proven thanks to evidence from animal models, tumor analysis, and *in vivo* analysis such as 1H-RM spectroscopy (56, 60), pituitary tumorigenesis in patients with an *SDHx* pathogenic variant does not seem to be systematically due to a somatic second hit according to the Knudson model (61). Moreover, the frequency of putative *SDHx* pathogenic variants in the general population, assessed by the frequency of loss of function variants in genome database like gnomAD, compared to the rarity of this condition questions the benefit of PitNET screening in carriers of *SDHx* mutations.

On the other hand, several patients have been reported with a *MAX* pathogenic or likely pathogenic variant and a NET other than pheochromocytoma in the literature. *MAX* codes for the MAX protein, a component of the MYC signaling pathway. The protein forms heterodimers with C-MYC via basic-helix-loop-helix zipper domain interactions. These heterodimers can then bind to target DNA sequences or E-BOX sequences to regulate transcription of genes involved in cell proliferation and cell growth. *MAX* behaves as a tumor suppressor gene. Germline and somatic *MAX* variants can result in PPGL (62). To date, at least seven patients with *MAX* variants and PitNET have been reported, six of whom had functional PitNETs (4 lactotroph PitNETs, 2 somatotroph PitNETs) (59, 63, 64).

Other NETs have been reported in patients carrying *MAX* mutations with or without PitNETs, including ganglioneuroma, ganglioneuroblastoma, adrenomedullary hyperplasia, pancreatic NETs, and parathyroid adenomas, but also nonendocrine tumors, including renal oncocytomas, renal carcinomas, breast carcinomas, and squamous cell tumors (62, 64–67). Nevertheless, the causal link between these non-PPGL tumors and *MAX* variants remains to be established.

The *CDC73* gene is also suspected to be implicated in MEN1 phenocopy, since the report of a patient with a clinical diagnosis of MEN1, based on the combined occurrence of normocalcemic PHPT at age 70 years, acromegaly diagnosed at age 22 years, and a pancreatic NET at age 70 years, harboring a heterozygous c.1138C>T p.(Leu380Phe) *CDC73* germline variant suspected to be pathogenic (68). Characterization of the pancreatic tumor confirmed the neuroendocrine origin of the neoplasm with positive immunostaining for chromogranin and glucagon. *CDC73* is a tumor suppressor gene. *CDC73* germline pathogenic variants are responsible for hyperparathyroidism-jaw tumor (HPT-JT) syndrome (OMIM 145001), which associates parathyroid adenoma or carcinoma, fibro-osseous jaw tumor, cystic kidney lesion, uterine tumors and in rare cases tumors of the thyroid, testis, and pituitary in a single patient (31, 69, 70). In a report by Lines et al. (68), the p.(Leu380Phe)*CDC73* variant was suspected of pathogenicity because the variant (i) occurred in a highly conserved residue, (ii) is involved in the interaction domain, (iii) is absent from 120,000 individuals in the gnomAD database, and (iv) is predicted to have a damaging effect by computational analysis. Concerning the involvement of this *CDC73* variant in pancreatic NETs, an RNA-Scope analysis showed a significant reduction in *CDC73* expression (13.5% (p<0.005)), compared to the peritumoral normal pancreas. However, this decreased expression was not shown by immunohistochemistry and questions the link between *CDC73* and the pancreatic tumor in this patient.

## 5 Gene-disease relationship assessment in the era of genomic medicine

Advances in genomic sequencing technology have led to the number of new gene-disease relationships rapidly expanding. However, the evidence supporting these claims varies widely, often without an accurate evaluation of genomic variations in a clinical setting. The NIH-funded Clinical Genome Resource (ClinGen) has developed a framework to define and evaluate the clinical validity of gene-disease pairs across a variety of Mendelian disorders (71). They first defined six classes to qualitatively describe the strength of evidence supporting a gene-disease association. For example, the evidence to support a causal role for a gene in a disease is considered as limited, (i) if there are fewer than three observations of variants that provide convincing evidence for disease causality, or (ii) if variants have been observed in probands, but none have sufficient evidence for disease causality, and (iii) if there is limited experimental data supporting the gene-disease association. In contrast, variants that disrupt function (such as truncating variants in tumor suppressor genes) and/or that are associated with other strong evidence in genetics or in population data (e.g. *de novo* occurrence, absence in large control cohorts such as the gnomAD database, strong linkage to a small genomic interval, etc.) are considered convincing evidence of disease causality. The authors developed a semiquantitative approach to evaluate both genetic and experimental evidence in a standardized manner that promotes consistent collection and weighting of evidence. Genetic evidence is evaluated based on case information: (i) *de novo* occurrence of the suspected variant, (ii) variants causing loss of function, (iii) evidence of segregation in one or more families, and (iv) case-control study data, provided that quality criteria are met, such as a sufficient number of cases and controls given the prevalence of the disease, case-control matching limiting bias and confounding factors (the same demographic information between cases and controls, the same phenotypical evaluation…), taking into account multiple testing for statistical significance, the use of methods for variant detection with equivalent analytical performance in cases and controls. For example, we can assign a stronger level of confidence for the pathogenicity of a *MAX* variant in a patient with multiple endocrine neoplasia if other candidate genes were ruled out, especially if genome-wide analysis was performed. Conversely, the finding of another pathogenic variant in a gene with a well-established gene-disease association decreases the confidence level. In 2021, Raygada et al. reported the case of a woman with an adrenocortical carcinoma associated with double germline mutations in *MSH2* and *RET* (72)*. MSH2* is one of the family of DNA mismatch repair genes, a group of tumor suppressor genes that are involved in Lynch syndrome, a syndrome that predisposes subjects to colorectal and endometrial cancers, as well as adrenocortical carcinoma. The patient carried the *RET* variant c.2410G>A, p.Val804Met is a well-known activating mutation involved in MEN2A with a moderate risk of MTC and an incidence of pheochromocytoma and primary hyperparathyroidism of less than 10%. She also carried a deleterious germline mutation in the *MSH2* gene c.211+1G>T, p.(?) affecting splicing, that was found with a loss of heterozygosity in the adrenal tumor, ruling out the potential involvement of the *RET* mutant in this tumor, and thus representing only an incidental finding.

Experimental evidence also needs also to be evaluated and ranked. Obviously, it is important that the function of the candidate gene product is consistent with the phenotype of an affected individual or has a similar function to another gene known to be involved in the same disease. For tumor suppressor genes, examining for the loss of protein expression by immunohistochemistry and/or the occurrence of a second hit in the tumor should be performed, as a minimum, to document the alteration of gene function. Nevertheless, this criterion is not sufficient to provide either a strong level of evidence or to definitively rule out a gene, because interpretation of the results may be complicated by potential pitfalls including an inappropriate target for immunostaining, a dose effect of protein expression, or because the second hit could be missed if it is an epigenetic event that cannot be detected by conventional sequencing. On the other hand, loss of heterozygosity can be due to large chromosome remodeling that fortuitously includes the candidate gene. For this reason, ideally, we should assess the alteration of function of the protein in human genetically-modified cells, and in animal or non-human cell-culture models with a similarly disrupted copy of the affected gene. The aim of these experiments is to observe a phenotype in these models that is consistent with the human disease state, and potentially rescuing the phenotype in cells derived from affected individuals or engineered equivalents through the addition of the wild-type gene product or correction by gene editing.

Over the past two decades, preclinical research has turned increasingly more to cultured spheroids, tumoroids and organoids to investigate tissue pathophysiology and responses to current and novel drugs therapies (73, 74). Organoids are heterogeneous self-organizing 3D aggregates that can recapitulate the structure, function, and thereby overall biological complexity of organs, mainly obtained after redifferentiation of induced pluripotent stem cells (iPSC) (75). Tumoroids are generated from patient tumor samples where the various cell types can aggregate *in vitro* and recreate the tumor microenvironment. Spheroids are similar, but typically arise from the aggregation of one cell type, such as immortalized cell lines. In 2022, Noltes et al. generated a patient-derived parathyroid organoid model from hyperplastic parathyroid gland biopsies and showed that the parathyroid organoid model recapitulated the tissue at the gene and protein levels and showed appropriate responses to different calcium concentrations and drugs (76). In 2023, Mallick et al. used genetically-engineered iPSC derived organoids to model the development of corticotroph PitNETs expressing *USP48* or *USP8* somatic mutations (73). Several groups have generated MEN1 –patient-derived iPSC, showing that it is possible to reprogram cells from patients with rare endocrine diseases (77, 78). These technologies could one day be used to test new candidate genes.

In parallel, large cohort studies must be undertaken to better understand the natural history, expressivity, and penetrance of rare endocrine diseases. Indeed, though MEN1 and MEN2 are well-characterized diseases (12, 14), there are relatively few large series describing the phenotype of patients carrying *CDC73*- or *MAX* mutations with follow-up data. Such epidemiological data would enable precise phenotypic characterization, including the assessment of tumor risk according to gene, age or variant, and finally would allow patients to be included in personalized precision medicine programs as well as allowing accurate genetic counseling to be proposed for families.

## 6 Conclusion

With advances in genomic sequencing technology, the number of reported variants and gene-disease relationships has rapidly expanded. Since not all sequencing technologies are universally available and individual laboratories may choose different strategies, physicians must be trained to understand the aims, limits, advantages, and pitfalls of genetic testing, so that patients with these rare diseases do not risk an incorrect diagnosis due to the failure to perform further genetic analyses. On the other hand, efforts must be made at the regional and national levels to establish molecular diagnostics networks to optimize genetic diagnosis in difficult cases. Variants in disease causing genes must be carefully and regularly evaluated according to the current state of the art data. In patients carrying known pathogenic variants, the occurrence of tumors outside the known tumor gene spectrum could reveal a new gene-tumor association or may be due to another genetic origin, including another genetic disease. In any case, experimental and epidemiological studies must be conducted into rare endocrine diseases to better characterize the links between known and new candidate genes and multiple endocrine and non-endocrine neoplasia in these patients.
